# Narcissistic Perfectionism does not lead to an increased perception of Academic Efficacy

**DOI:** 10.1192/j.eurpsy.2023.2062

**Published:** 2023-07-19

**Authors:** M. J. Brito, C. Seco, A. T. Pereira, A. Macedo

**Affiliations:** 1 Coimbra University Hospital Centre; 2 Institute of Medical Psychology; 3 University of Coimbra, Coimbra, Portugal; 4 Department of Psychiatry, Coimbra University Hospital Centre, Coimbra

## Abstract

**Introduction:**

The relationship between narcissism and burnout has been explored in the literature with somewhat inconsistent findings. Though most studies have found a positive correlation between Narcissism and Burnout, some have failed to establish a significant link between the two, while others have even reported a protective role of narcissism against burnout.

In our previous work regarding the link between perfectionism and student burnout, we found that when using the Big Three model of Perfectionism, Narcissistic Perfectionism had only a weak connection to burnout, requiring full mediation by low-self compassion.

We hypothesized that this might be due to an exaggerated sense of Academic Efficacy in Narcissistic Perfectionists, which would compensate for some of the emotional exhaustion and depersonalization brought upon by their efforts to gain the admiration of others.

**Objectives:**

To investigate the link between Narcissistic Perfectionism and Academic Efficacy, and its impact on burnout levels.

**Methods:**

A sample of 1080 students from healthcare-related courses (80,7% females; mean age=21.13±3.023; range: 17-41) filled in an online questionnaire including, among others, the Portuguese Version of BIG3-SF and MBI-SS. Correlational analysis was performed.

**Results:**

Contrary to our initial theory, Narcissistic Perfectionism did not significantly correlate with Academic Efficacy (r=0.011, p=0.728), although it had significant correlations with the other burnout dimensions and total burnout score.

**Conclusions:**

This work disproved our initial hypothesis, suggesting that narcissistic perfectionism may be associated with other nefarious dimensions that cancel out the effects of grandiosity and inflated self-esteem on the perception of academic efficacy. This negative finding could possibly be further explored by using a psychometric instrument that differentiates between maladaptive and adaptive facets of narcissism.

**Disclosure of Interest:**

None Declared

